# Synchronous Cervical Adenocarcinoma and Ovarian Serous Adenocarcinoma—A Case Report and Literature Review

**DOI:** 10.3390/medicina56040152

**Published:** 2020-03-29

**Authors:** Nicolae Bacalbasa, Irina Cecilia Balescu, Camelia Diaconu, Simona Dima, Laura Iliescu, Mihaela Vilcu, Alexandru Filipescu, Ioana Halmaciu, Dragos Cretoiu, Iulian Brezean

**Affiliations:** 1 Department of Obstetrics and Gynecology, “Carol Davila” University of Medicine and Pharmacy, 020021 Bucharest, Romania; nicolae_bacalbasa@yahoo.ro (N.B.); alexandru.filipescu@gmail.ro (A.F.); 2 Department of Obstetrics and Gynecology, “I. Cantacuzino” Clinical Hospital, 030167 Bucharest, Romania; 3 Department of Visceral Surgery, Center of Excellence in Translational Medicine “Fundeni” Clinical Institute, 022328 Bucharest, Romania; simona.dima@gmail.ro; 4 Department of Surgery, “Ponderas” Academic Hospital, 021188 Bucharest, Romania; 5 Department of Surgery, “Carol Davila” University of Medicine and Pharmacy, 020021 Bucharest, Romania; 6 Department of Internal Medicine, “Floreasca” Clinical Emergency Hospital, 105402, Bucharest, Romania; drcameliadiaconu@gmail.com; 7 Department of Internal Medicine, “Carol Davila” University of Medicine and Pharmacy, 020021 Bucharest, Romania; laura.iliescu@gmail.ro; 8 Department of Internal Medicine, “Fundeni” Clinical Institute, 022328 Bucharest, Romania; 9 Department of Visceral Surgery, “Carol Davila” University of Medicine and Pharmacy, 020021 Bucharest, Romania; mihaela.vilcu@gmail.ro (M.V.); iulian.brezean@gmail.ro (I.B.); 10 Department of Visceral Surgery, “I. Cantacuzino” Clinical Hospital, 030167 Bucharest, Romania; 11 Department of Obstetrics and Gynecology, “Elias” Emergency Hospital, 105402 Bucharest, Romania; 12 Department of Anatomy, “George Emil Palade” University of Medicine, Pharmacy, Science and Technology, 540139 Târgu Mureș, Romania; ioana.halmaciu@gmail.ro; 13 “Alessandrescu-Rusescu” National Institute of Mother and Child Health, Fetal Medicine Excellence Research Center, 020395 Bucharest, Romania; dragos.cretoiu@gmail.ro; 14 Department of Cell and Molecular Biology and Histology, “Carol Davila” University of Medicine and Pharmacy, 020021 Bucharest, Romania

**Keywords:** synchronous malignancies, cervical adenocarcinoma, serous ovarian adenocarcinoma

## Abstract

*Background/Aim:* Synchronous gynecological malignancies are rarely encountered, and most often these cases are represented by synchronous ovarian and endometrial cancer. The aim of this paper is to present the case of a 53-year-old patient who was diagnosed with synchronous cervical and ovarian cancer. *Case presentation:* The patient had been initially investigated for vaginal bleeding and was submitted to a biopsy confirming the presence of a cervical adenocarcinoma. Once the diagnostic of malignancy was confirmed, the patient was submitted to a computed tomography which revealed the presence of large abdominal tumoral nodules of peritoneal carcinomatosis and was submitted to palliative chemotherapy with poor response. Eighteen months later she developed intestinal obstruction and was submitted to surgery. At that moment, synchronous ovarian and cervical tumors were diagnosed. Total radical hysterectomy with bilateral adnexectomy, pelvic and para-aortic lymph node dissection, omentectomy, and pelvic peritonectomy was performed; in the meantime, the histopathological studies confirmed the presence of two synchronous malignancies. *Conclusion*: Although synchronous lesions are rarely encountered, this eventuality should not be omitted. In such cases, surgery should be taken in consideration and the intent of radicality should regard both lesions.

## 1. Introduction

Synchronous gynecological malignancies can rarely be encountered in patients with gynecological tract tumors, the most commonly encountered lesions being represented by ovarian and endometrial cancer [1,2,3]. As for the association of cervical and ovarian cancer, this situation has been rarely reported, the most common association being of human papillomavirus (HPV)-induced cervical cancer and mucinous ovarian adenocarcinoma; only few cases have been reported so far [4,5,6]. The aim of the paper is to present the case of a patient who was initially diagnosed with advanced stage mucinous adenocarcinoma of the uterine cervix and presumed peritoneal carcinomatosis who was diagnosed at the time of surgery with synchronous cervical and ovarian carcinoma.

## 2. Case Presentation

The study was approved by the local ethics committee (11/27.01.2020) and the patient signed an informed consent form. The 53-year-old postmenopausal woman with no significant family oncological background was initially investigated for postmenopausal vaginal bleeding, diffuse pelvic pain, and weight loss. At that moment, vaginal examination revealed the presence of a cervical tumor which was biopsied.

The histopathological studies revealed the presence of a moderately differentiated cervical adenocarcinoma, so the patient was submitted to a pelvic magnetic resonance imaging and to a thoracic and abdominal computed tomography, which revealed the presence of large abdominal masses of peritoneal carcinomatosis. In the meantime, biological parameters did not reveal any significant modifications—the serum levels of cancer antigen 125 (CA125), human epididymis 4 (HE4), and carcinoembryonic antigen (CEA) reported minimal modification (CA125 = 76 U/mL, HE4 = 43 U/mL, CEA = 25 ng/mL). Therefore, the diagnostic for that moment was of cervical adenocarcinoma with peritoneal metastases; hence, the patient was submitted to platinum-based chemotherapy with palliative intent; however, at six months follow-up the imagistic studies revealed no significant response, and the patient became refractory to this treatment and decided to interrupt the oncological treatment. Therefore, due to the lack of compliance partially induced by the poor response to treatment, the patient was lost from follow up for more than a year.

**Figure 1:**
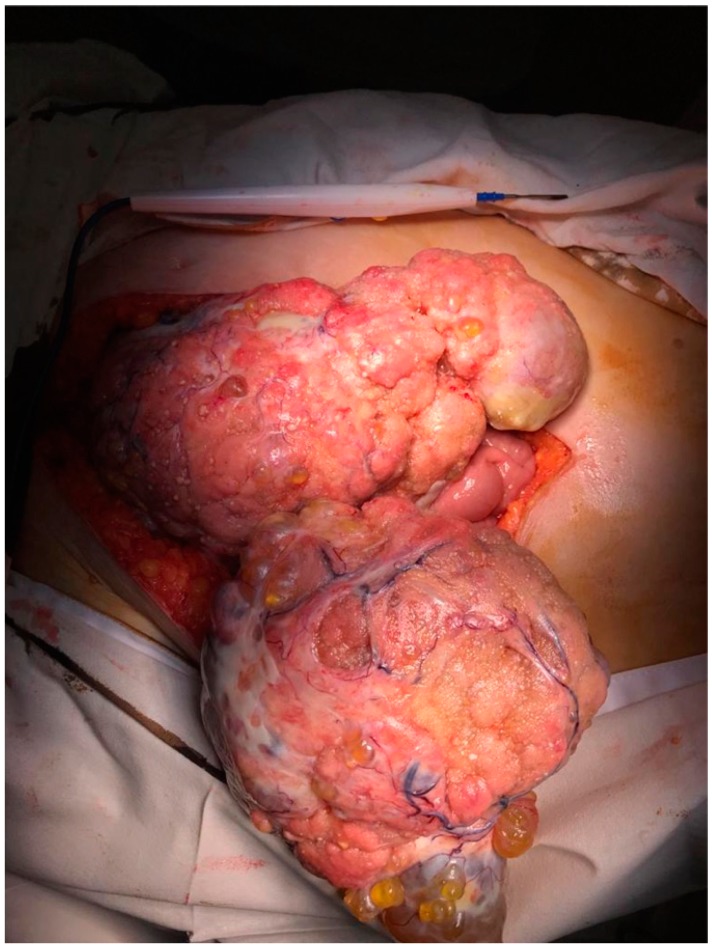
Initial intraoperative aspect—large nodules of peritoneal carcinomatosis.

**Figure 2:**
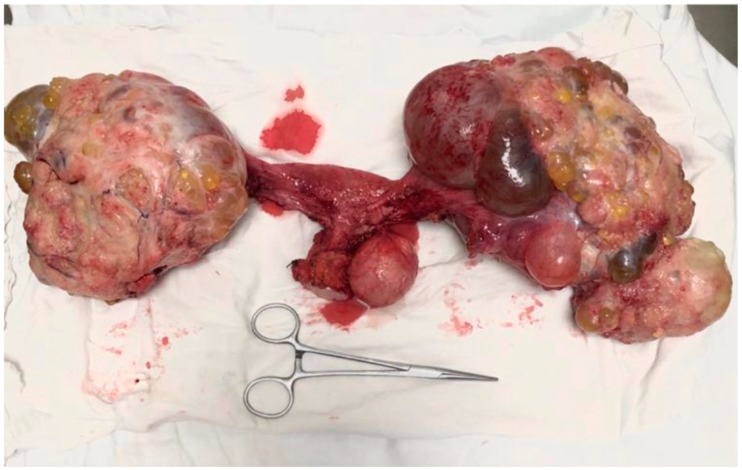
The final aspect—the specimen of total radical hysterectomy with bilateral adnexectomy and excision of the nodules of peritoneal carcinomatosis.

However, eighteen months later she came back due to the presence of diffuse abdominal pain and intestinal obstructive syndrome. The patient was submitted to surgery in order to treat the intestinal obstruction; at the time of surgery bilateral ovarian tumors were identified in association with peritoneal nodules of carcinomatosis and with the already known cervical tumor. The intraoperative frozen section demonstrated the presence of a serous ovarian adenocarcinoma and confirmed the fact that the peritoneal lesions had an ovarian origin; therefore, she was submitted to surgery with curative intent; in this respect, a total radical hysterectomy with bilateral adnexectomy, pelvic and para-aortic lymph node dissection, omentectomy, and pelvic peritonectomy were performed (Figure 1 and Figure 2).

The final histopathological findings confirmed the presence of a moderately differentiated mucinous cervical adenocarcinoma, in association with well differentiated ovarian serous adenocarcinoma with peritoneal involvement; in the meantime, three of the 21 pelvic lymph nodes presented tumoral involvement. The final staging was of stage IIIC ovarian adenocarcinoma (cancer has spread to the peritoneum and the cancer in the peritoneum is larger than 2 centimeters and/or cancer has spread to lymph nodes in the abdomen) and stage IIA cervical cancer (cancer has spread to the uterus and/or fallopian tubes (the long slender tubes through which eggs pass from the ovaries to the uterus); according to the World Health Organization (WHO) 2014 classification, the ovarian lesion was classified as a low grade serous carcinoma, while the cervical lesion was classified as a not otherwise specified mucinous carcinoma [7]. Postoperatively she was confined to the oncology service in order to be submitted to adjuvant consolidation chemotherapy. In the meantime, after establishing the final histopathological diagnostic, BRCA1/2 (BReast CAncer genes 1 and 2) testing was performed; however, none of these mutations were encountered.

## 3. Discussion

Synchronous gynecological malignancies have been encountered in up to 2% of women diagnosed with any kind of gynecological cancers; however, the most commonly encountered association consists of ovarian and endometrial cancer [3,4,5,6]. As for the association between ovarian and cervical cancer, isolated cases have been reported so far: Therefore, in a study conducted by Turkish authors on a period of 20 years, there were only five patients diagnosed with synchronous ovarian and cervical cancer [8]. In a more recent study conducted by Young et al. on 20 patients with synchronous gynecological malignancies, a single patient was diagnosed with synchronous ovarian and cervical carcinoma [6].

In such cases the most important question which is raised is if the two lesions are real synchronous, distinct malignancies, or if one of them is the metastasis of the other tumor. This fact can be clearly ruled out in cases in which the tumors exhibit different subtypes, as well as in cases in which areas of normal parenchyma can be evidenced between the two lesions. Therefore, in our case, the presence of two different histopathological subtypes (serous ovarian adenocarcinoma and mucinous cervical adenocarcinoma) sustained the hypothesis of synchronous lesions in an indubitable manner.

Uterine cervix adenocarcinoma represents the second most common histopathological subtype of cervical cancer after squamous cell carcinoma, ranging for up to 20% of cases with cervical cancer. However, in the last decades, the incidence of cervical adenocarcinoma increased, especially due to the fact that screening tests are less effective for this histopathological subtype [9,10,11]. The low efficacy of detection of this histopathological subtype during routine evaluation is rather related to the endocervical development of these tumors; therefore, an important number of cases are diagnosed at a more advanced stage of the disease, when local extension or distant metastases transform the patient into a candidate for palliative treatment [12,13]. Moreover, this histopathological subtype has been initially considered as being associated with a poorer outcome when compared to squamous cell carcinoma [14]. However, more recent and larger studies came to demonstrate that association of targeted therapies such as monoclonal antibodies, angiogenic kinases by tyrosine kinase inhibitors, immune checkpoint programmed cell death 1, and T-lymphocyte-associated molecule-4 inhibitors might significantly improve the outcomes of these patients, especially when used in the setting of advanced or recurrent disease [15]. In the meantime, in cases diagnosed with cervical adenocarcinoma, the risk of lymph node metastases development is rather related to the presence of stromal invasion and lymph vascular invasion than to the dimensions of the tumor [13,14]. Another important feature of this histopathological subtype is represented by the capacity to develop distant metastases at the level of the ovaries and lymph nodes, when compared to squamous cell carcinoma [16,17,18,19]. However, in our case, the fact that the ovarian tumors proved to have a serous component, while the cervical adenocarcinoma proved to be a mucinous tumor, enabled us to consider that the two malignancies were synchronous and not metastatic lesions. Moreover, the degree of differentiation of the ovarian tumor probably explained the absence of a negative impact of the long period of time in which the patient did not undergo any treatment; in the meantime, the patient’s decision to interrupt the treatment against the medical recommendation and her poor compliance with the initial treatment can be explained by the fact that under this treatment only minor improvement of the general status was observed.

As for the type of treatment of cervical adenocarcinoma, it is usually similar to the one for squamous cell cervical cancer [20,21,22]. However, association of monoclonal antibodies in the setting of advanced stage or recurrent disease provided a significant improvement of the long-term outcomes of these cases [15]. These data were also sustained by the study conducted between 2004 and 2014 at the Royal Marsden Hospital, which demonstrated that second line therapy in the setting of advanced and recurrent cervical cancer is associated with poor rates of response; moreover, the authors underlined the necessity of exploring the effectiveness of novel targeted agents and immunotherapy in such cases. Data related in this study are particularly important due to the fact that different and aggressive biological subtypes such as clear cell, adenocarcinoma, and even neuroendocrine uterine cervix carcinomas have been included [23]. As for the cases diagnosed in early stages of the disease, fertility sparing surgery should not be taken into consideration, due to the more aggressive biological behavior of this histopathological subtype.

Mucinous adenocarcinomas of the uterine cervix are an even scarcer biological subtype, being considered as a particular subgroup since 2014; one of the largest studies which came to investigate the prognostic factors in patients with mucinous adenocarcinoma of uterine cervix has been recently published in the *Journal of Gynecology Obstetrics and Human Reproduction* and included 82 cases diagnosed with cervical adenocarcinomas, 21 of them being diagnosed with the mucinous subtype [24]. Among these cases, the mean age was of 42 years, while the most commonly encountered symptoms were represented by vaginal bleeding, followed by mucinous discharge; moreover, only 72% of cases presented a modified smear test. As for the type of performed treatment, it consisted of total radical hysterectomy with bilateral adnexectomy and sentinel node biopsy in cases diagnosed in early stages of disease and complete lymph node dissection in more advanced stages; in the meantime, surgery was followed by adjuvant oncological treatment. After a mean follow-up of 30 months, 13 cases were alive and free of disease, one was alive with disease, three were dead of disease, one was dead due to other causes, and the remaining three cases were lost from follow-up [24]. These data come to suggest that although this histopathological subtype is significantly scarcer, it benefits from the same type of oncological and surgical treatment when compared to squamous cell carcinoma. As for the case we presented, it was treated in a similar manner once the hypothesis of peritoneal carcinomatosis with cervical origin was excluded; therefore, the patient benefitted from a radical hysterectomy with extended pelvic and para-aortic lymph node dissection.

However, the correct and complete diagnostic in our case was delayed with almost two years, especially due to the fact that the ovarian malignancy was not associated with increased levels of CA125 and HE4, being a non-secretory tumor; this aspect in association with the poor compliance of the patient with the proposed treatment led to a delay of the treatment with curative intent.

When it comes to the utility of CA125 in ovarian cancer diagnostic, attention should be focused on the fact that only half of patients are in early stages of the disease and up to 90% of cases diagnosed in advanced stages present increased levels, the remaining 10% of cases presenting normal or minimal increase, of the serum levels of CA125 [25]. In the meantime, in order to maximize the rates of detection of ovarian cancer even in cases in which serum levels of CA125 could not give a diagnostic clue, HE4 has been proposed; therefore, it is estimated that two thirds of patients diagnosed with early or advanced stage of the disease will exhibit increased levels of HE4. Moreover, certain studies have shown that one third of patients with normal levels of CA125 and ovarian cancer will exhibit increased levels of HE4; however, in our case, neither CA125 nor HE4 did report any suggestive modification for the diagnostic of ovarian cancer. In this respect, the treatment was initially tailored according to the suspicion of diagnostic of cervical adenocarcinoma and peritoneal carcinomatosis with cervical origin. Other serum markers which might increase the rates of preoperative diagnostic of ovarian cancer in postmenopausal women are represented by alpha fetoprotein and β-human chorionic gonadotrophin; however, these parameters seem to be beneficial, especially in cases diagnosed with particular histopathological subtypes, such as germ cell ovarian tumors [26]. In this respect, novel tumor markers and scores are still needed in order to provide a better preoperative identification of such pathologies.

## 4. Conclusions

Although rarely seen, synchronous cervical and ovarian cancer might be encountered and might benefit from surgery with curative intent. However, in the case we reported, the presence of a non-secretory ovarian adenocarcinoma, even in the presence of large nodules of peritoneal carcinomatosis in association with the indubitable diagnostic of cervical adenocarcinoma, led to a confusing initial diagnostic. Moreover, the fluctuant evolution of this patient was also caused by the initial poor compliance of the patient with treatment and follow-up. However, the final diagnostic was established at the time of surgery for obstructive syndrome, which revealed the presence of two different lesions and which offered the chance to the patient to be submitted to surgery with curative intent, radical procedures being performed for both ovarian and cervical malignant lesions.
